# Using a resident-led patient safety quality council to educate future QI leaders

**DOI:** 10.1080/10872981.2020.1855699

**Published:** 2020-12-02

**Authors:** Nirvani Goolsarran, Kevin Zarrabi, Christine Garcia

**Affiliations:** a Department of Medicine, Stony Brook University Hospital, Stony Brook, NY, USA; b Department of Medicine, University of Pittsburgh Medical Center, Pittsburgh, PA, USA

**Keywords:** Patient safety, quality improvement, medical education, graduate medical education

## Abstract

Resident-led councils represent an important initiative to involve trainees in patient safety, but little is known about how to create and sustain one of these councils. We evaluated the impact of a resident-led patient safety council in an internal medicine residency program. We assessed change in resident perception of safety issues over 3 years, scholarship activities, and behavioral choices to participate or lead patient safety activities after residency.

The Stony Brook Internal Medicine Residency Program formed the Patient Safety and Quality Council (PSQC) in 2014, consisting of fifteen peer-nominated residents serving a three-year term. Surveys were distributed annually from 2014 to 2017 to measure resident council members’ perception of patient safety. The number of safety-related abstract/publications were tracked during and one year after graduation. Additionally, graduates from the council were surveyed to assess the influence of the council on post residency involvement and leadership in safety activities.

A total of 18 residents have participated in the council from 2014 to 2017. Overall, resident perception of safety culture improved. A total of 17/18 (94%) PSQC resident members demonstrated scholarship activities in safety during residency: 8/18 (44%) were engaged in an independent Quality Improvement (QI) project, 5/18 (27%) achieved a quality improvement leadership role post residency. A total of 15 of 18 (83%) recent graduates suggest that involvement with the safety council during residency fostered future involvement in patient safety.

Implementation of a resident-led safety council can help to improve the safety culture, generate scholarly activities, and encourage continued participation in patient safety after graduation.

## Introduction

Historically, resident participation in departmental and institutional-based patient safety and Quality Improvement (QI) has been limited [1]. To address this need, many residency programs and hospitals across the country have taken various approaches to address QI education. Some have developed QI curricula; while others have adopted resident-run safety councils, which are becoming more and more common [2]. Many studies demonstrate the success of resident-led councils in terms of process improvements and impact on patient care [3]. Although these reports are of paramount importance, to our knowledge there are limited studies on the impact on scholarship productivity, which represents dissemination of QI work, as well as influence of the council on post residency engagement in safety and QI.

The call for physician leaders who are skilled in QI and patient safety is rising across the nation [4]. Although the impact of a resident-led safety council on post-residency engagement and career choices in safety is not well documented, there are numerous studies that demonstrate self-reported positive influence on future career interests. Resident-led council models encourage continued participation in patient safety after residency graduation [1].

We implemented a resident-led safety council that generated scholarly activities and encouraged continued participation in patient safety after graduation, using the Kirkpatrick evaluation model for educational intervention to demonstrate both learning satisfaction and behavioral influence.

## Methods

### Council creation and mission

Stony Brook University Hospital is an academic medical center and serves as the tertiary care center of Suffolk County, New York. The internal medicine residency program consists of approximately 96 trainees from Post-Graduate Year (PGY) one through three. A resident-led Internal Medicine Residency Patient Safety and Quality Council (PSQC) was formed in August 2014. The main objectives and mission of the PSQC include: to serve as ambassadors of patient safety and QI; and to address safety concerns brought up by the residents. At its inception, the scholarly goals related to quality and safety of the council were discussed and agreed upon. Specifically, the council’s goals included: participate in patient safety/quality day, generate abstracts and publications from the quality initiatives encountered, and hopefully motivate council members to become involved in QI after residency completion.

## Structure of the committee

Fifteen residents from all post-graduate years 1 to 3 (five from each year) of training were peer-nominated and recruited to join the newly formed PSQC. The goal was to form a small committee with adequate representation from each resident PGY level. Recognizing that physicians alone cannot adequately address safety, interdisciplinary faculty representatives volunteered to also serve as the leaders of the PSQC. The council members consisted of a patient safety officer from the institution, a quality nurse, pharmacist, informatics personnel and various faculty members. Meetings took place on a monthly basis for one-hour duration on the last Wednesday of the month at 6 pm. PSQC residents determine a monthly agenda based on reported safety issues brought up by the medicine residents through either in-person communication or via a PSQC medicine email inbox. Safety or quality issues are identified by residents during their routine practice on the wards or in the clinic setting. Based on the issue, respective department representatives are invited to help address the specific safety issue. The members of the council conduct group-based QI projects using the Plan-Do-Study-Act (PDSA) methodology as they tackle the safety issues. SMART (Specific, Measureable, Achievable, Relevant, Time-bound) aim statements are developed during the meeting and group members continue to work on QI projects on their own time with faculty mentorship outside of the meetings.

## Assessment of councils impact

A survey was created to assess council members’ perceptions of patient safety within the institution. The surveys were distributed on the first day of council inception and were redistributed at the end of each academic year (June 2015, 2016, and 2017). An excel spreadsheet was developed to track abstract presentations and publications generated from the PSQC initiatives. An additional survey was emailed to the PSQC graduates who had completed their training in 2018 to assess the impact of the council on post-graduation engagement and leadership in patient safety and QI. The impact of the council on process improvements was specifically assessed and published in a prior study [4].

## Results

**Figure 1.**
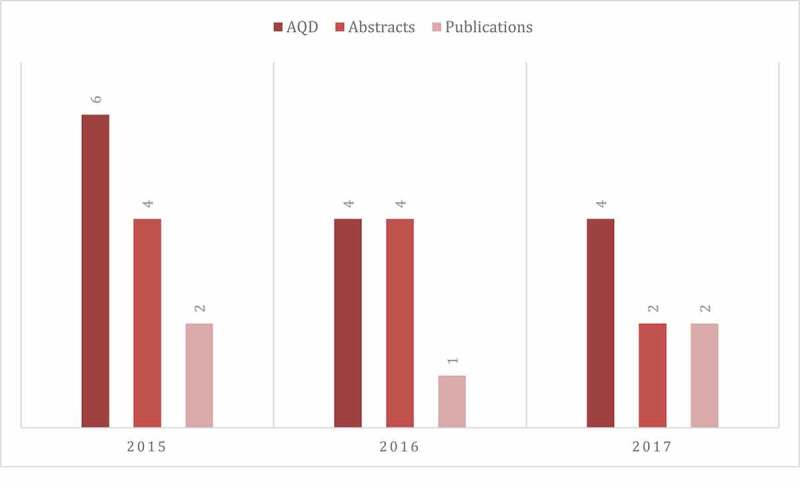
Mean number of abstracts and publications per resident, by year. AQD, Annual Quality Day

EMR, electronic medical record; ER, emergency room; MRI, magnetic resonance imaging; MICU, medical intensive care unit; CAUTI, catheter-associated urinary tract infection; ACGME, accreditation council of graduate medical education.

Results from the annual survey demonstrate that the PSQC has had a positive impact on the culture of safety within the residency program (Table 1). Council membership grew over time to a total number of 18 residents who served for a three-year period and graduated in 2017. New resident members were recruited each year based on resident nomination. Overall, a total of 17/18 (94%) PSQC resident members demonstrated scholarship activities in safety during residency as abstract/poster presentations at Stony Brook Annual Quality Day, as well as in local and national meetings. Of those, 8/18 (27%) publications were in peer-reviewed QI journals (Figure 1). Additionally, 8/18 (44%) were engaged in an independent QI project, 5/18 (27%) achieved a QI leadership role post residency. A total of 15 of 18 (83%) recent graduates suggest that involvement with the safety council during residency fostered future involvement in patient safety. Table 2 lists examples of quality improvement projects and outcomes that resulted from the council’s work from 2015 to 2017.

**Table 1.** Annual survey results on patient safety attitudes

Residents agree or strongly agree with the statement, *n* (%)					
Statement	2014, *n* = 15	2015, *n* = 15	2016, *n* = 14	2017, *n* = 14	2018, *n* = 14
Patient safety is a priority in this program	12 (80)	14 (93)	14 (100)	14 (100)	14 (100)
The just culture is emphasized in this program	13 (86)	13 (86)	13 (93)	14 (100)	14 (100)
My level of confidence increased in developing quality improvement projects	–	12 (80)	14 (100)	14 (100)	14 (100)
Participation in safety activities can lead to scholarly projects (posters, abstracts, publications	11 (73)	14 (93)	14 (100)	14 (100)	14 (100)
I think I can make a difference in patient safety at my institution	13 (86)	15 (100)	13 (93)	14 (100)	14 (100)

**Table 2.** Examples of quality improvement projects and outcomes

QI Project that resulted in abstracts/publications	Outcomes of the project
Standardization and sustainability of the hand-off process (published)	Project resulted in the use of a standardized hand-off process for all residents; this was integrated in the EMR [5]
Addressing delays patient during direct admission process (published)	Project resulted in creation of a triage tool and communication device for admission that bypass the ER [6]
Improve MRI safety screening (published)	Project resulted in improved screening for safe MRI using an automated screening tool in the EMR [7]
Education on team work and communication with nursing staff (published)	Project resulted in implementation of simulation education to enable resident trainees to work in teams with nursing students [8]
Development of an escalation protocol for EMS transport (presented as abstracts to Stony Brook Annual Quality Day 2016, AAMC Integrating Quality meeting 2017)	Project resulted in allowing EMS team to escalate care triage to MICU while in route to hospital
Initiative to decrease the incidence of CAUTI infection (presented as abstract to Stony Brook Annual Quality Day, 2015)	Project resulted in automated pager communication to the provider as a reminder to remove foley catheters
Improving compliance with medication reconciliation presented as abstract to ACGME Annual Meeting, 2015	Project resulted in education reminders and checklist for completion of medication reconciliation

## Discussion

Resident-led PSQC is extremely valuable in not only promoting resident engagement in process improvement but can effectively serve as a venue for QI scholarship and dissemination in a residency program. Residents at our institution completed a formalized longitudinal QI curriculum during their ambulatory weeks as part of residency training. The QI projects conducted by the PSQC residents supplemented their training and further developed their motivation to continue QI initiatives. Using the Kirkpatrick evaluation model for educational intervention, we used survey data on the perception of the council to demonstrate learning satisfaction, and data on recent graduate involvement in safety roles as a marker of behavioral influence.

Our study has several limitations. This study particularly focuses on the committee’s success as a function of QI scholarship and influence on QI engagement and leadership after graduation from residency, relying on self-reported resident perception of the safety culture of the residency program. We also did not measure prior data for comparison of QI scholarly output prior to the inception of the PSQC; therefore, we cannot make a strong conclusion that there is abstract productivity as a direct result of the PSQC. Further studies can use a better approach to assess the impact of the council on scholarship productivity by comparing the scholarly output of the PSQC group versus a non-PSQC group.

We noted an initial rapid rise followed by an apparent decreasing trend in mean number of abstract and publication from 2015 to 2017. A surge of enthusiasm and interest at the inception of the council followed by a slight decline is a potential explanation for the observed trend. This highlights the importance of sustainability planning (e.g., keeping members engaged in relevant projects) to maintain outcome achievements in resident QI projects. Finally, we studied a single departmental, single institution intervention with a small group of residents.

We believe that the success of PSQC forum is reliant on three important factors: 1) peer selection of residents; this promotes identification of safety champions who are accountable for representing and promoting safety for their respective peers, 2) the support and endorsement of the Residency Program Director/Vice Chair of Education, and the Department Chair; they provided a small budget for dinners during the monthly meetings, 3) using scholarship (abstracts and publications) as a dissemination strategy to motivate residents to conduct spontaneous and voluntary QI.

## Conclusion

Implementation of a resident-led safety council can help to improve the safety culture, generate safety/QI scholarly activities, and encourage continued participation in patient safety after graduation. More importantly, a PSQC has the potential to generate future QI leaders that can continue to generate independent QI projects even after transition to a new role and institution.
